# Transcriptome Analysis Reveals Key Genes and Pathways Associated with the Petal Color Formation in Cabbage (*Brassica oleracea* L. var. *capitata*)

**DOI:** 10.3390/ijms23126656

**Published:** 2022-06-15

**Authors:** Bin Zhang, Jiao Wang, Li Chen, Wenjing Ren, Fengqing Han, Zhiyuan Fang, Limei Yang, Mu Zhuang, Honghao Lv, Yong Wang, Jialei Ji, Yangyong Zhang

**Affiliations:** Key Laboratory of Biology and Genetic Improvement of Horticultural Crops, Institute of Vegetables and Flowers, Chinese Academy of Agricultural Sciences, Ministry of Agriculture, #12 Zhong Guan Cun Nandajie Street, Beijing 100081, China; 13126720352@163.com (B.Z.); wangjiaoynl@163.com (J.W.); 18205480752@163.com (L.C.); 17863805323@163.com (W.R.); feng857142@163.com (F.H.); fangzhiyuan@caas.cn (Z.F.); yanglimei@caas.cn (L.Y.); zhuangmu@caas.cn (M.Z.); lvhonghao@caas.cn (H.L.); wangyong@caas.cn (Y.W.); jijialei@caas.cn (J.J.)

**Keywords:** cabbage, petal color, transcriptomic, *BoCCD4*, carotenoid, expression analysis

## Abstract

Petal color is an important agronomic trait in cabbage (*Brassica oleracea* L. var. *capitata*). Although the key gene *BoCCD4* has been functionally characterized, the underlying molecular regulatory mechanism of petal color formation in cabbage is still unclear. In this study, we applied the transcriptome analysis of yellow petals from the cabbage inbred line YL-1 and white petals from the Chinese kale inbred line A192-1 and the *BoCCD4*-overexpressing transgenic line YF-2 (YL-1 background), which revealed 1928 DEGs common to both the A192-1 vs. YL-1 and the YL-1 vs. YF-2 comparison groups. One key enzyme-encoding gene, *BoAAO3*, and two key TF-encoding genes, *Bo2g151880* (WRKY) and *Bo3g024180* (SBP), related to carotenoid biosynthesis were significantly up-regulated in both the A192-1 and YF-2 petals, which was consistent with the expression pattern of *BoCCD4*. We speculate that these key genes may interact with *BoCCD4* to jointly regulate carotenoid biosynthesis in cabbage petals. This study provides new insights into the molecular regulatory mechanism underlying petal color formation in cabbage.

## 1. Introduction

Carotenoids are important pigments in the photosynthetic system and play essential roles in human nutrition and health because of their provitamin A and antioxidant property. Carotenoids are precursors of abscisic acid (ABA) and other hormones that regulate plant growth. Carotenoids usually accumulate in flowers and fruits and provide yellow, orange and red colors that attract pollinators for plant reproduction. In addition, carotenoids are used in the cosmetic, food and pharmaceutical industries [1,2,3,4,5,6].

In recent years, transcriptome analysis has been performed on many species by RNA sequencing (RNA-seq) to reveal the different mechanisms governing pigment biosynthesis. In *Brassica rapa*, the carotenoid biosynthesis-related genes and the paralogues of each carotenoid biosynthesis-related gene showed different expression patterns across flower, stem, leaf, root and silique tissues according to transcriptome analysis [7]. In *Brassica napus*, the transcriptome analysis of white and yellow petals revealed 20 significantly differentially expressed genes (DEGs) involved in the carotenoid metabolic pathway, among which *BnNCED4b* was markedly up-regulated in white petals [8]. In papaya, transcriptome analysis showed that the expression levels of key carotenoid biosynthesis pathway-related genes were different between yellow peel and red flesh tissues [3]. In tobacco, pink and white flower transcriptome analysis showed that anthocyanin biosynthesis-related structural genes and genes encoding some bHLH and MYB transcription factors (TFs) were strongly down-regulated in white flowers [9]. In cabbage, 43 DEGs were identified in the *ygl-1* gene mapping region in which recombination was suppressed, among which three DEGs may be strongly associated with the yellow-green leaf phenotype [10].

The flowers of Chinese kale are mostly white, while cabbage flowers are typically yellow. Petal color variations are usually caused by mutations of the gene involved in the carotenoid or anthocyanin metabolic pathways, which alters the content of pigments, resulting in petals with different colors. In *B. napus* and Chinese kale, the insertion of a CACTA-like transposable element disrupts the function of *BnaC3.CCD4* and *BoCCD4.2*, causing the petal color to change from white to yellow [5,11]. In *Osmanthus fragrans* and *B. napus*, the expression of *OfCCD4* and *BnNCED4b* can be regulated by the transcription factors *OfWRKY3* and *BnWRKY22*, associated with the white petal formation, respectively [8,12]. In *Mimulus lewisii*, the loss-of-function of TF *R2R3-MYB* leads to the down-regulation of all carotenoid biosynthetic genes and to reduced carotenoid content in flowers [13]. In our previous studies, the carotenoid cleavage dioxygenase 4 (*BoCCD4*) gene, which is responsible for white petal color formation, was functionally identified in *Brassica oleracea* [14]. However, the *BoCCD4* interactors and molecular mechanism underlying petal color formation in *B. oleracea* are unclear.

Here, we performed transcriptome profiling of yellow petals from the YL-1 cabbage inbred line and white petals from the A192-1 Chinese kale inbred line and the YF-2 *BoCCD4*-overexpressing transgenic line (YL-1 background). The findings lay a foundation for revealing the molecular regulatory mechanism underlying white/yellow petal color formation in cabbage.

## 2. Results

### 2.1. RNA-Seq and DEG Analysis of A192-1, YL-1 and YF-2 Petals

**Figure 1:**
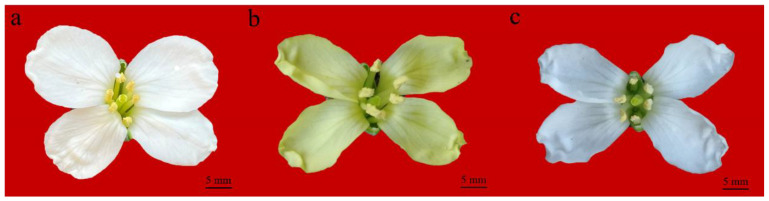
Phenotypes of A192-1, YL-1 and YF-2. (**a**) A192-1 with white petals. (**b**) YL-1 with yellow petals. (**c**) YF-2 with white petals. Bar = 5 mm.

Six cDNA libraries of petal samples of A192-1 (white petals’ Figure 1a), YL-1 (yellow petals’ Figure 1b) and YF-2 (white petals’ Figure 1c) were sequenced to obtain DEGs. After removing adaptor sequences, low-quality reads and ambiguous reads, 88.72 (A192-1), 87.60 (YL-1) and 76.57 (YF-2) million clean reads were obtained, and all the Q30 values were >92%. The clean reads were then mapped to the *B. oleracea* TO1000 reference genome (http://plants.ensembl.org/Brassica_oleracea/Info/Index (accessed on 26 January 2022)). All the total mapping percentages were >88% (Table 1), and the density distribution and boxplots of all the genes exhibited similar patterns among the six samples, indicating that the transcriptome sequencing data were reliable for further analysis (Supplementary Figure S1).

**Figure 2:**
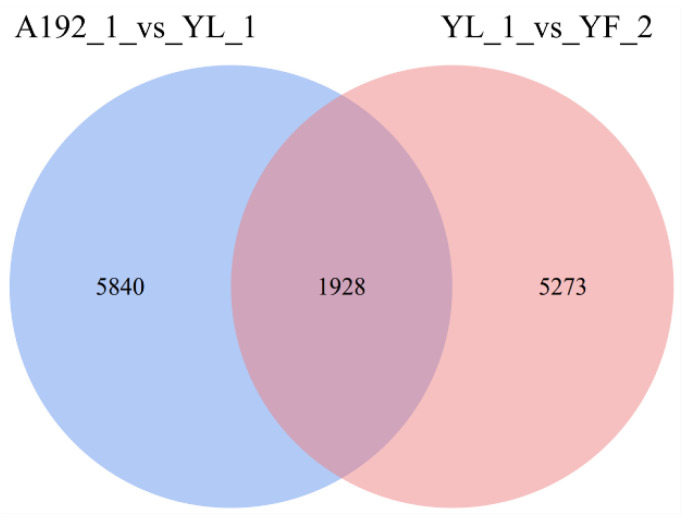
Venn diagram showing the numbers of DEGs in the A192-1 vs. YL-1 and YL-1 vs. YF-2 comparison groups.

In total, 7768 (3493 up- and 4275 down-regulated) and 7201 (4229 up- and 2972 down-regulated) DEGs were detected in the A192-1 vs. YL-1 and YL-1 vs. YF-2 comparison groups, respectively (Supplementary Figure S2). A Venn diagram analysis revealed that 1928 DEGs were common to the A192-1 vs. YL-1 and YL-1 vs. YF-2 comparisons (Figure 2), including 1026 up-regulated and 902 down-regulated genes in the A192-1 vs. YL-1 comparison group and 964 up-regulated and 964 down-regulated genes in the YL-1 vs. YF-2 group (Supplementary Table S2), indicating that these genes may be strongly related to petal color formation in cabbage.

### 2.2. GO and KEGG Pathway Enrichment Analysis of DEGs

**Figure 3:**
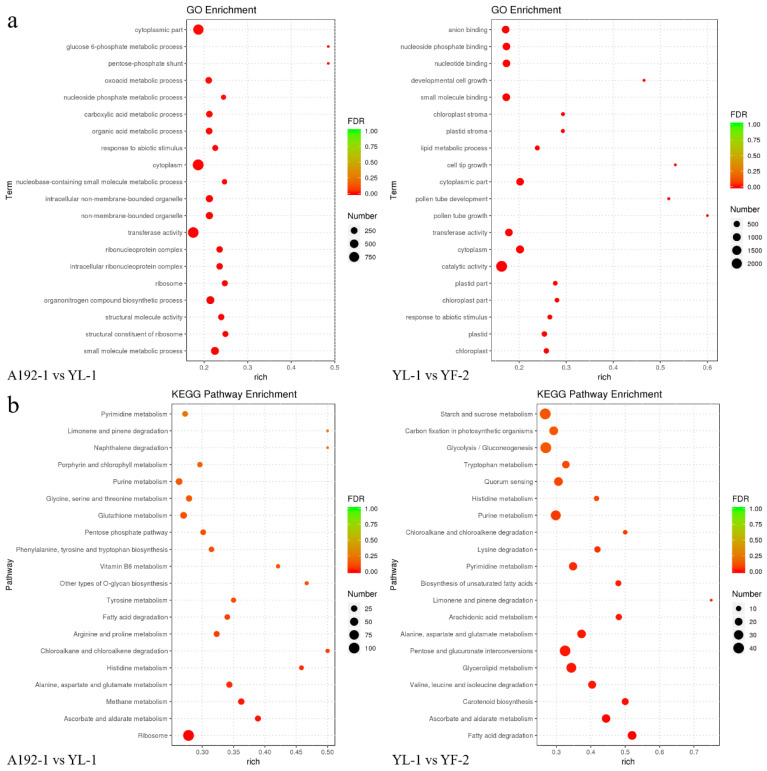
Top 20 enriched GO terms and KEGG pathways in the A192-1 vs. YL-1 and YL-1 vs. YF-2 comparison groups. (**a**) GO terms for the three comparison groups. The X-axis represents the rich factor, and the Y-axis represents the GO terms. (**b**) KEGG pathways for the three comparison groups. The X-axis represents the rich factor, and the Y-axis represents the pathway terms.

The DEGs were classified into three GO categories: the biological process (BP), cellular component (CC) and molecular function (MF) categories. The top 20 enriched terms were identified in each comparison group. The most significantly enriched terms were small-molecule metabolic process (BP), structural constituent of ribosome (MF), chloroplast (CC) and plastid (CC) in the A192-1 vs. YL-1 and YL-1 vs. YF-2 comparison groups (Figure 3a). KEGG analysis was subsequently performed to uncover the important biological functions of the DEGs, and the top 20 enriched pathways were identified in each comparison group. Ascorbate and aldarate metabolism was the most significantly enriched pathway in both the A192-1 vs. YL-1 and YL-1 vs. YF-2 comparison groups. In addition, ribosome and methane metabolism, fatty acid degradation and carotenoid biosynthesis were the most highly enriched pathways in the A192-1 vs. YL-1 and YL-1 vs. YF-2 comparison groups (Figure 3b).

### 2.3. Expression Analysis of DEGs Involved in the Carotenoid Biosynthetic Pathway

**Figure 4:**
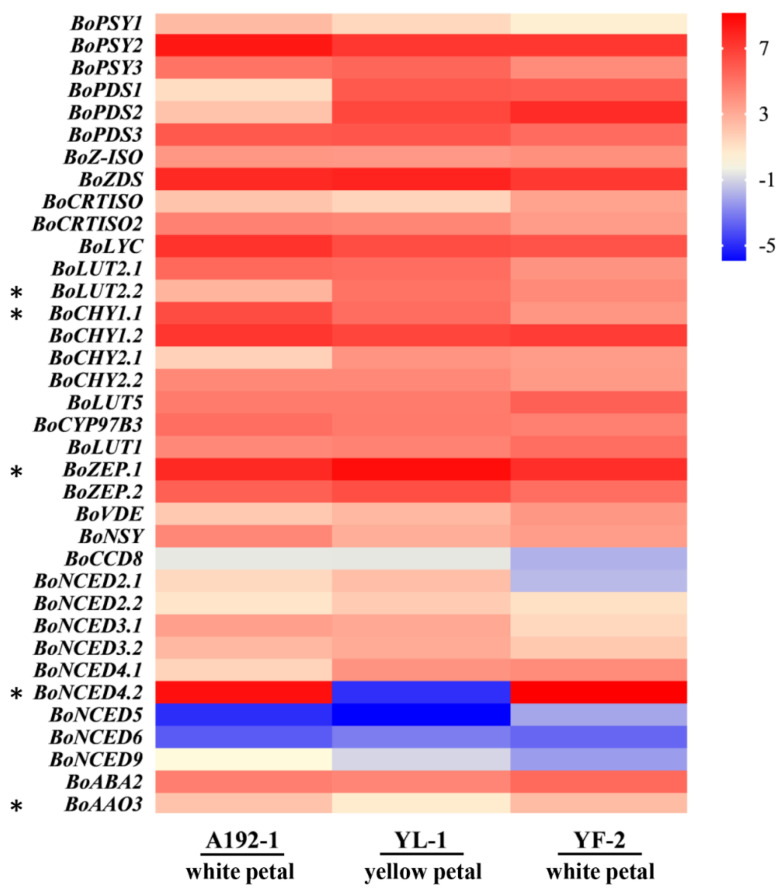
Heatmap of gene expression patterns involved in the carotenoid biosynthetic pathway in A192−1, YL−1 and YF−2. The heatmap was constructed according to the FPKM average values. The color scale represents the highest and lowest levels of expression, and the rows and columns in the heatmap represent samples and genes, respectively. The asterisks represent significant differences in both A192−1 and YF−2 petals (*p* < 0.05).

The carotenoid biosynthetic pathway has been thoroughly characterized [15,16,17]. A total of 33 homologous genes involved in the carotenoid biosynthetic pathway were identified in *B. oleracea*, of which 10 (6 up- and 4 down-regulated) and 18 (15 up- and 3 down-regulated) were significantly differentially expressed in the A192-1 vs. YL-1 and YF-2 vs. YL-1 comparison groups, respectively (Figure 4; Supplementary Table S3). Among these DEGs, only *BoLUT2.2* and *BoZEP.1* were significantly down-regulated in both the A192-1 and YF-2 petals, and only *BoNCED4.2* (*BoCCD4*) and *BoAAO3* were significantly up-regulated in both the A192-1 and YF-2 petals; this was especially true for *BoCCD4*, which showed an abnormally high expression in the white petals (Figure 4), indicating that *BoCCD4* may interact with *BoAAO3* to jointly regulate carotenoid biosynthesis in cabbage petals.

### 2.4. Identification of Key Transcription Factors Related to the Carotenoid Metabolic Pathway

A previous study reported that members of the MYB, SBP, bHLH, NAC, WRKY, HD-ZIP and MADS-box TF families are the major regulators of carotenoid metabolism-related genes [18]. The top 20 significantly differentially expressed genes encoding TFs were then analyzed in the YL-1 vs. A192-1 and YL-1 vs. YF-2 comparison groups. Among these TF-encoding genes, only *Bo2g151880* (WRKY) and *Bo3g024180* (SBP) were significantly up-regulated in both the A192-1 and YF-2 petals in a manner that was consistent with the expression pattern of *BoCCD4* (Supplementary Table S4), indicating that these genes may regulate carotenoid metabolism by regulating *BoCCD4* gene expression.

### 2.5. qRT-PCR Validation of Key DEGs Related to the Carotenoid Biosynthetic Pathway

**Figure 5:**
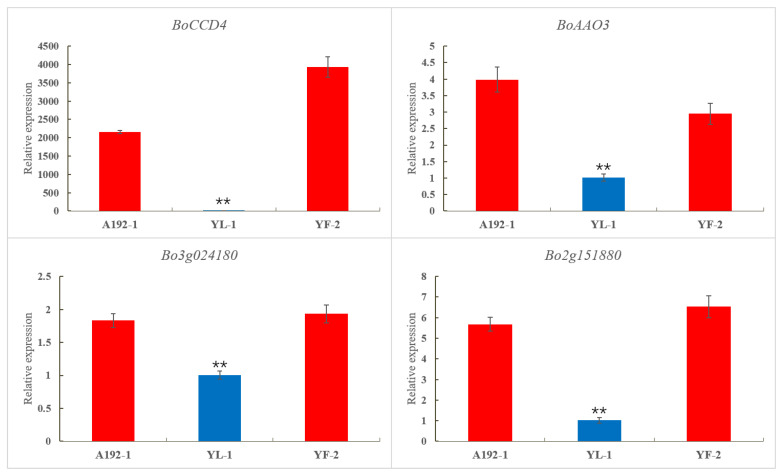
Transcript levels of six key DEGs related to carotenoid metabolism in A192-1, YL-1 and YF-2. The error bars represent the standard errors of three biological replicates. The asterisks represent significant differences (*p* < 0.01).

The expression patterns of two key enzymes, *BoCCD4* and *BoAAO3*, and two key TFs, *Bo2g151880* and *Bo3g024180*, related to the carotenoid metabolic pathway in 11-192, YL-1 and YF-2 petals were verified via qRT–PCR. All the genes showed significantly higher expression levels in the white petals compared with the yellow petals, which is consistent with the transcriptome results; this was especially true for the BoCCD4 gene, whose expression was barely detected in the yellow petals (Figure 5).

## 3. Discussion

In *B. napus*, Jia et al. (2021) identified 1209 DEGs in WP vs. ZS11 petals at four different stages by transcriptome analysis, including 20 DEGs involved in the carotenoid metabolism pathway. In our study, 1928 DEGs were identified in A192-1 vs. YL-1 and YL-1 vs. YF-2 petals, and only five DEGs were involved in the carotenoid biosynthetic pathway (Supplementary Table S2). Among these five DEGs, *BoNCED4.2* (*BoCCD4*) and *BoAAO3* were significantly up-regulated in both the A192-1 and YF-2 petals. The *BoCCD4* gene responsible for petal color formation was barely expressed in the yellow petals, which is consistent with the findings of previous studies [8,11,14]. We speculated that the cabbage petals will show varying degrees of color change from yellow to white with the change in the *BoCCD4* expression level. Importantly, the *BoAAO3* gene (a homologue of *Arabidopsis ATAAO3*), which mediates the conversion of 9-cis-epoxycarotenoids to ABA [7,8,19], showed a significantly high expression in the white petals that was consistent with the expression pattern of *BoCCD4*. In addition, the interaction mode of AAO3 was predicted with STRING (https://cn.string-db.org/cgi/input?sessionId=bSaM2E7MJvrB&input_page_show_search=on (accessed on 19 March 2022)), which showed that AAO3 may interact with NCED3 and ABAs in the carotenoid metabolic pathway (Supplementary Figure S3). However, the *BoNCED3s* had no significant expression difference in the A192-1 vs. YL-1 comparison group and were significantly down-regulated in the YF-2 petals. The *BoABA2* gene showed no significant expression difference in either the A192-1 vs. YL-1 or YF-2 vs. YL-1 comparison groups (Supplementary Table S3). Taken together, these findings indicated that *BoAAO3* may interact with BoCCD4 to regulate carotenoid degradation in cabbage petals.

Transcription factors play a crucial role in regulating carotenoid biosynthesis. In *B. napus*, Jia et al. (2021) identified six TFs that were significantly up-regulated in white petals at all four stages. In our study, only two TFs, *Bo3g024180* (a homologue of *Arabidopsis SPL13*) and *Bo2g151880* (a homologue of *Arabidopsis WRKY74*), were identified as being dramatically up-regulated in both the A192-1 and YF-2 petals. *SPL13* was reported to regulate flowering time and shoot branching in *Medicago sativa* [20], and *OsSPL13* was identified as regulating grain length and seed yield in rice [21]. In *Osmanthus fragrans*, *OfWRKY3* positively regulates *OfCCD4* gene expression by binding to the W-box palindrome motif present in the *OfCCD4* promoter [12]. Jia et al. (2021) suggested that *BnWRKY22* in *B. napus* may act as an upstream TF regulating *BnNCED4b* expression. Therefore, we considered that *Bo2g151880* (*WRKY74*) may be a strong regulator of *BoCCD4* in the regulation of carotenoid metabolism.

MYB transcription factors carry out important functions in plants. In *Arabidopsis*, *AtMYB2* can function as a transcriptional activator in the ABA signaling pathway [22]. In papaya, *CpMYB1* and *CpMYB2* have a function in fruit ripening and carotenoid accumulation by regulating cell-wall degradation and carotenoid biosynthesis-related genes [23]. In *Actinidia deliciosa*, *MYB7* plays a role in regulating carotenoid and chlorophyll accumulation in fruit [24]. In *Mimulus lewisii*, *R2R3-MYB* plays a critical role in regulating flower carotenoid pigmentation [13]. In our study, six MYB TFs, *Bo6g122640*, *Bo9g003750*, *Bo7g033260*, *Bo4g046000*, *Bo5g008270* and *Bo7g011290*, were identified in the top 20 significantly up-regulated TFs in the A192-1 or YF-2 petals (Supplementary Table S4). These *MYBs* may interact with the *BoCCD4* promoter to regulate carotenoid biosynthesis, providing further insights into the *BoCCD4*-mediated regulatory pathways underlying petal color formation in cabbage.

## 4. Conclusions

In this study, one key enzyme, *BoAAO3*, and two key transcription factors, *Bo2g151880* (WRKY) and *Bo3g024180* (SBP), were identified as potential interactors with *BoCCD4* to coregulate carotenoid biosynthesis by transcriptome analysis and qRT–PCR validation. This study lays a foundation for revealing the molecular regulatory mechanism underlying white/yellow petal color formation in cabbage.

## 5. Materials and Methods

### 5.1. Plant Materials

A192-1 is a Chinese kale inbred line with white petals, YL-1 is a cabbage inbred line with yellow petals and YF-2 is a *BoCCD4*-overexpressing transgenic line (YL-1 background) with white petals. All the plant materials used in the present study were grown in a greenhouse (25 ± 2 °C) under a 16 h light/8 h dark photoperiod at the Institute of Vegetables and Flowers, Chinese Academy of Agriculture Sciences (IVFCAAS, Beijing, China). During the flowering stage, petal samples of A192-1, YL-1 and YF-2 were collected from five individuals, respectively. Two biological replicates were performed per sample.

### 5.2. RNA Extraction and Sequencing

The total RNA from all of the collected samples was extracted using a TIANGEN RNAprep Pure Plant Kit (Tiangen Biotech Co., Ltd., Beijing, China) according to the manufacturer’s instructions. The RNA purity and quality were determined using a spectrophotometer (BioDrop, UK) and agarose gel electrophoresis. A total of six cDNA libraries were constructed and subsequently sequenced with an Illumina Hi-Seq 2000 platform by Biomarker Technologies Co., Ltd. (Beijing, China).

### 5.3. Data Analysis

The clean reads were aligned to the *B. oleracea* TO1000 reference genome (http://plants.ensembl.org/Brassica_oleracea/Info/Index (accessed on 26 January 2022)) by HISAT [25,26]. The DEGs were identified by DEGseq, with the selection criteria |log_2_(fold-change)| > 1 and the q-value < 0.05 for significant differential expression. Gene Ontology (GO) functional enrichment analysis and Kyoto Encyclopedia of Genes and Genomes (KEGG) pathway enrichment analysis were performed on the DEGs using the clusterProfiler software.

### 5.4. qRT–PCR Validation

First-strand cDNA was synthesized using a FastKing RT Kit (TIANGEN) following the manufacturer’s instructions. qRT–PCR was carried out using a TransStart Top Green qPCR SuperMix Kit (TransGen Biotech) on a CFX96 Real-Time System (Bio-Rad). All the experiments were performed for three biological and three technical replicates. The relative expression levels of the genes were calculated by the 2^−ΔΔCt^ method [27]. *B. oleracea actin* was used as the internal reference gene. The qRT–PCR primers used are shown in Supplementary Table S1.

## Figures and Tables

**Table 1:** Overview of the transcriptome sequencing dataset.

Samples	A192-1-1	A192-1-2	YL-1-1	YL-1-2	YF-2-1	YF-2-2
Raw reads	49,470,348	47,645,834	45,147,382	49,510,444	41,944,966	40,739,642
Clean bases	45,118,684	43,601,476	41,801,266	45,799,878	38,837,852	37,736,246
Q20 (%)	97.55	96.63	97.55	97.43	96.80	97.10
Q30 (%)	94.38	92.55	94.24	93.98	92.41	92.98
Total mapped reads (%)	92.16	91.38	89.74	89.68	88.51	88.35
Uniquely mapped reads (%)	96.49	97.12	97.41	97.28	95.95	96.42

## Data Availability

All the data generated or analyzed in this study are included in this published article and its supplementary information files. The *B. oleracea* TO1000 reference genome used in this study can be found at http://plants.ensembl.org/Brassica_oleracea/Info/Index (accessed on 26 January 2022). The NCBI protein database can be found at https://www.ncbi.nlm.nih.gov/ (accessed on 12 February 2022). The *A. thaliana* genome can be found at https://www.arabidopsis.org/ (accessed on 18 February 2022). All these databases are publicly accessible.

## Supplementary Materials

They are available online at https://www.mdpi.com/article/10.3390/ijms23126656/s1.
