# Supplementary material for: Transcriptome Analysis Reveals Key Genes and Pathways Associated with the Petal Color Formation in Cabbage (Brassica oleracea L. var. capitata)
Source: Int J Mol Sci. 2022 Jun 15;23(12):6656. doi: 10.3390/ijms23126656 (PMC9224331; doi:10.3390/ijms23126656)
Supplement: Supplementary file 1 [file ijms-23-06656-s001.zip › Supplementary Materials.pdf]

Supplementary Information

Figure S1. Distribution of the expression levels of all genes in the six petal samples.

Figure S2. Volcano plots of DEGs in three comparison groups. The red dots represent significantly up-regulated DEGs, and the green dots represent significantly down-regulated DEGs. |log2(fold-change)| > 1 and q-value < 0.05 were used as criteria for statistical significance.

Figure S3. The putative interaction networks of AAO3.

Table S1. Sequences of primer used for qRT–PCR in this study.

Table S2. Information on 1928 DEGs common in the A192-1 vs. YL-1 and YL-1 vs. YF-2 comparison groups.

Table S3. Expression patterns of thirty-three homologous genes involved in the carotenoid biosynthetic pathway in the A192-1 vs. YL-1 and YL-1 vs. YF-2 comparison groups.

Table S4. Expression patterns of the top 20 significantly differentially expressed TFs in the A192-1 vs. YL-1 and YL-1 vs. YF-2 comparison groups.
