# Supplementary figures and images for: Transcriptome Analysis Reveals Key Genes and Pathways Associated with the Petal Color Formation in Cabbage (Brassica oleracea L. var. capitata)
Source: Int J Mol Sci. 2022 Jun 15;23(12):6656. doi: 10.3390/ijms23126656 (PMC9224331; doi:10.3390/ijms23126656)

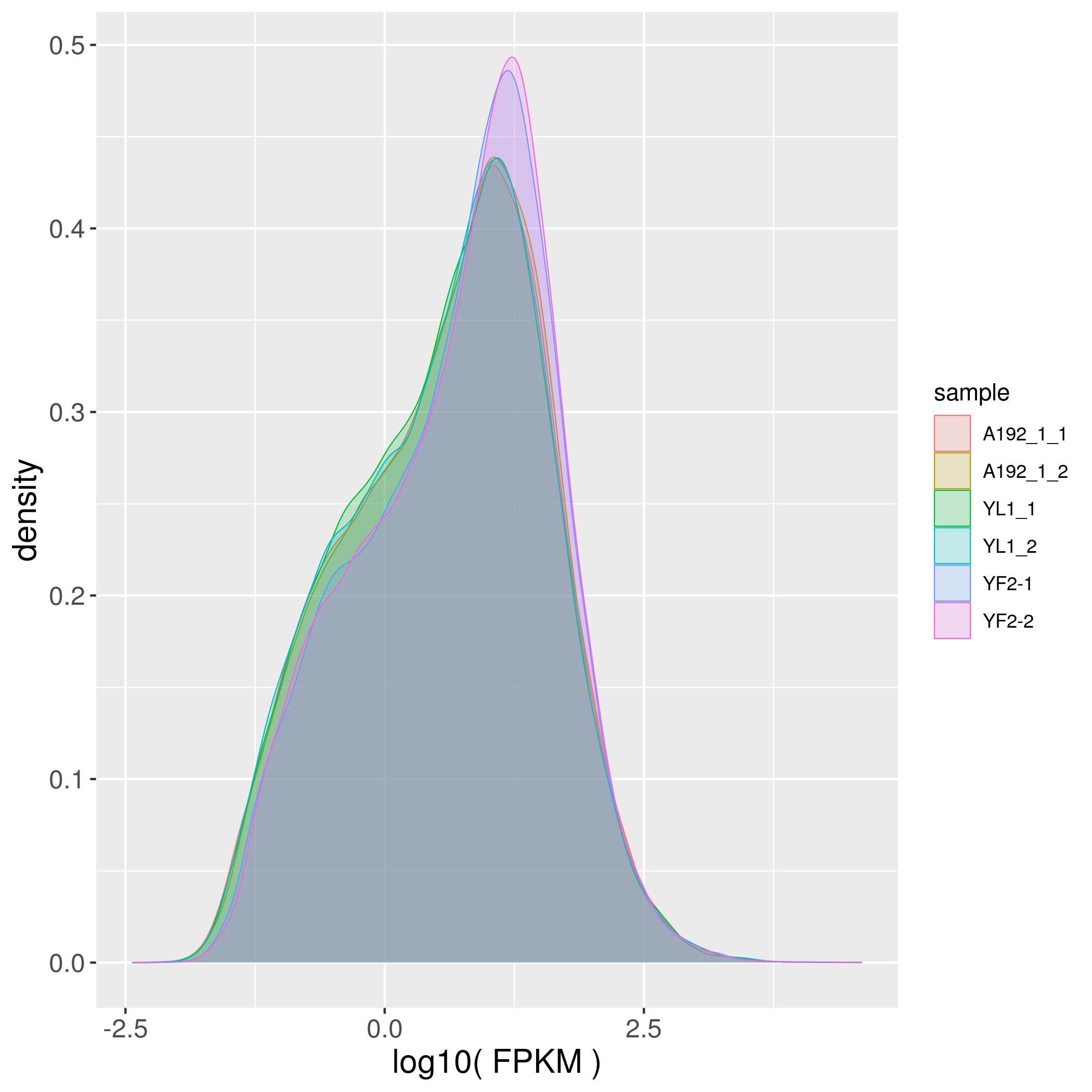

Supplement: Supplementary file 1 [file ijms-23-06656-s001.zip › Figure S1.png]

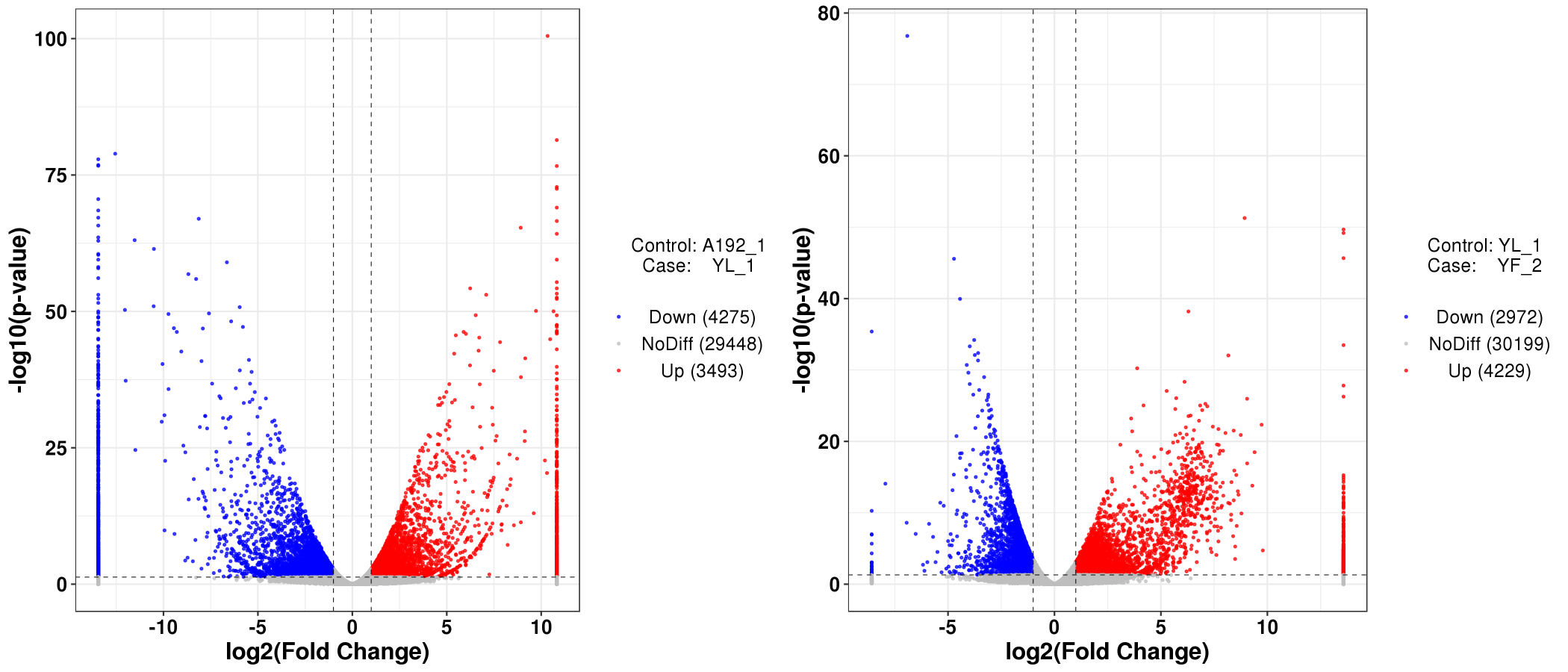

Supplement: Supplementary file 1 [file ijms-23-06656-s001.zip › Figure S2.png]

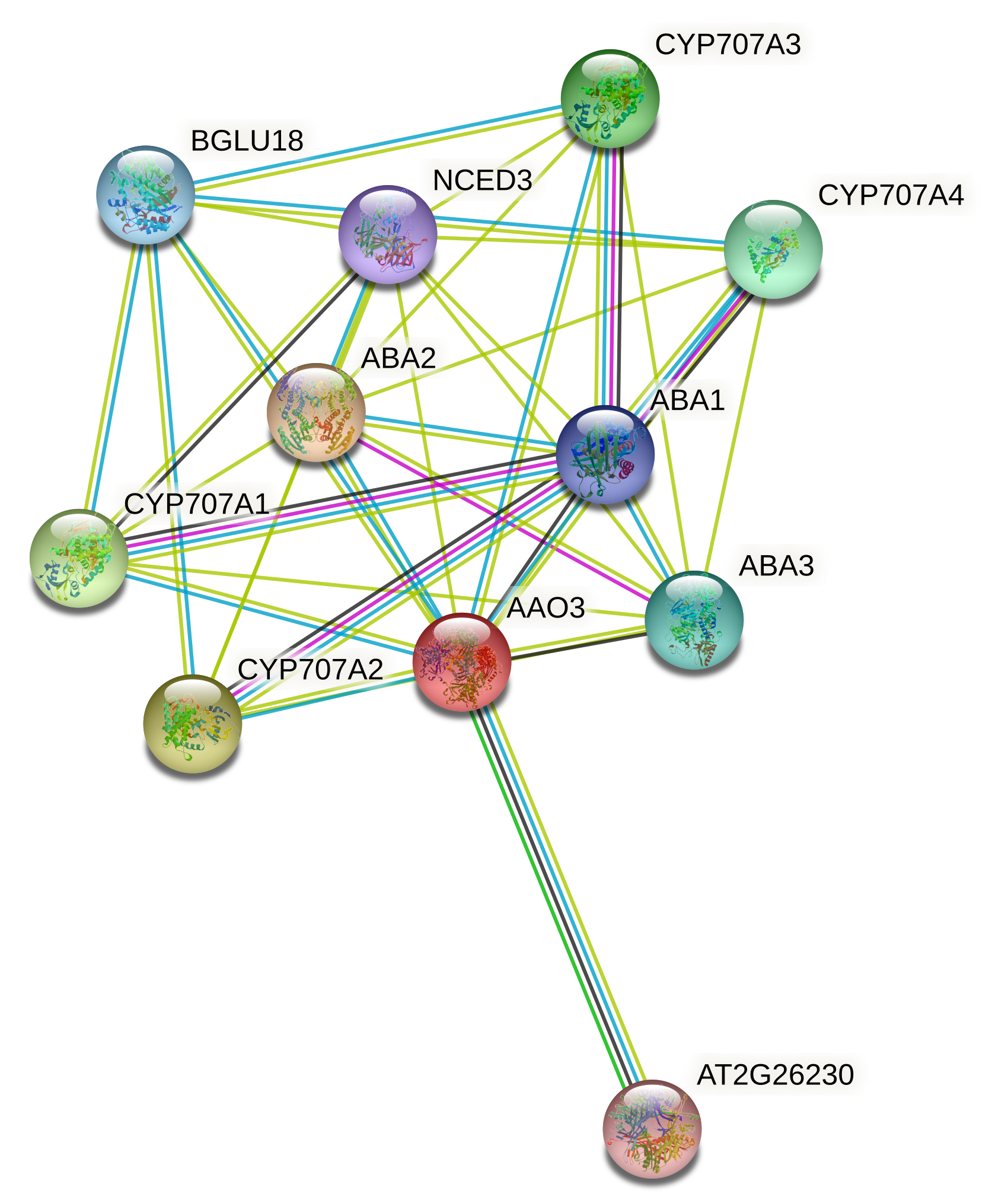

Supplement: Supplementary file 1 [file ijms-23-06656-s001.zip › Figure S3.png]
